# Melatonin Protects Cultured Tobacco Cells against Lead-Induced Cell Death *via* Inhibition of Cytochrome c Translocation

**DOI:** 10.3389/fpls.2017.01560

**Published:** 2017-09-14

**Authors:** Agnieszka Kobylińska, Russel J. Reiter, Malgorzata M. Posmyk

**Affiliations:** ^1^Laboratory of Plant Ecophysiology, Faculty of Biology and Environmental Protection, University of Lodz Lodz, Poland; ^2^Department of Cellular and Structural Biology, UT Health Science Center, San Antonio TX, United States

**Keywords:** BY-2 tobacco cells, cytochrome c, DNA fragmentation, melatonin, programmed cell death

## Abstract

Melatonin was discovered in plants more than two decades ago and, especially in the last decade, it has captured the interests of plant biologists. Beyond its possible participation in photoperiod processes and its role as a direct free radical scavenger as well as an indirect antioxidant, melatonin is also involved in plant defense strategies/reactions. However, the mechanisms that this indoleamine activates to improve plant stress tolerance still require identification and clarification. In the present report, the ability of exogenous melatonin to protect *Nicotiana tabacum* L. line Bright Yellow 2 (BY-2) suspension cells against the toxic exposure to lead was examined. Studies related to cell proliferation and viability, DNA fragmentation, possible translocation of cytochrome c from mitochondria to cytosol, cell morphology after fluorescence staining and also the *in situ* accumulation of superoxide radicals measured *via* the nitro blue tetrazolium reducing test, were conducted. This work establishes a novel finding by correcting the inhibition of release of mitochondrial ctytocrome c in to the cytoplasm with the high accumulation of superoxide radicals. The results show that pretreatment with 200 nm of melatonin protected tobacco cells from DNA damage caused by lead. Melatonin, as an efficacious antioxidant, limited superoxide radical accumulation as well as cytochrome c release thereby, it likely prevents the activation of the cascade of processes leading to cell death. Fluorescence staining with acridine orange and ethidium bromide documented that lead-stressed cells additionally treated with melatonin displayed intact nuclei. The results revealed that melatonin at proper dosage could significantly increase BY-2 cell proliferation and protected them against death. It was proved that melatonin could function as an effective priming agent to promote survival of tobacco cells under harmful lead-induced stress conditions.

## Introduction

In their natural environment, plants are exposed to many different biotic and abiotic stresses. Among various stressors, heavy metals, especially lead (Pb), are major environmental pollutants, particularly in areas with high anthropogenic pressure (Gill, 2014) and its accumulation has adverse effects on plant growth and crop productivity. Pb is phytotoxic and found in dust, fumes, mists, vapors and in soil as minerals (PbCO, PbS, PbSO_4_) (Nicholls and Mal, 2003). Although the level of heavy metals in agricultural soil is normally very low, the repeated use of phosphate fertilizers over long periods may cause dangerously high concentrations of some of these toxins (Gill, 2014). Pb is taken up *via* roots along with water, or it can be absorbed from the air *via* shoots and foliage (Fahr et al., 2013). Unfortunately, plant roots are not selective and absorb Pb with other minerals where accumulates. In a number of species, high Pb levels cause abnormal plant morphology, reduced plant growth and finally it induces cell death (Pourrut et al., 2012). Toxic Pb concentrations inhibit the activity of key enzymes, e.g., acid phosphatase, esterases, peroxidases, malic dehydrogenase, by reacting with their sulfhydryl groups. Moreover, Pb contributes to water imbalance, alterations in cell membrane permeability and it limits mineral nutrition. Pb excess also induces oxidative stress in tissues by increased reactive oxygen species (ROS) generation. Simultaneously, Pb provokes DNA damage, gene mutations, protein oxidation, lipid peroxidation and finally it promotes signal transduction cascades that promote cell death (Wierzbicka, 1999; Gill, 2014).

Programmed cell death (PCD) is an indispensable process for animals and plant development. In plant systems, PCD falls within two broad categories, environmentally induced and developmentally regulated cell death. Environmentally induced PCD is usually a consequence of external factors including heat shock (Vacca et al., 2006; Lord and Gunawardena, 2012), cold (Lei et al., 2004), pathogen infection leading to a hypersensitivity response (HR) (Mur et al., 2008; Pietrowska et al., 2015) and death caused by heavy metals (Iakimova et al., 2007; Iwase et al., 2014). PCD is an event displayed by many different organisms throughout evolution; however, despite the enormous evolutionary distance across organisms there are some common features including: increased formation of vesicles, cytoplasmic condensation, nuclear condensation, DNA laddering and translocation of cytochrome c (Cyt c) from mitochondria to the cytosol (Isbat et al., 2009; Martínez-Fábregas et al., 2014). In plant cells, Cyt c release occurs during PCD and is a result of many stimuli such as menadione, D-mannose, heat or ROS (Sun et al., 1999; Stein and Hansen, 1999; Tiwari et al., 2002; Vacca et al., 2004).

Petrosillo et al. (2003) documented that mitochondrial-induced ROS production promotes Cyt c release from mitochondria by a two-step process, including dissociation of Cyt c from cardiolipin, followed by permeabilization of the outer membrane, probably by interaction with voltage dependent anion channels. However, the function of cytoplasmic Cyt c is still controversial since Vacca et al. (2006) found that Cyt c release depended on ROS production, but it may not trigger PCD. Furthermore, after Cyt c translocation, caspase-like proteases inactivate it, leading to Cyt c degradation *en route* to PCD (Vacca et al., 2006). However, data of Martínez-Fábregas et al. (2014) indicated that extra-mitochondrial Cyt c had a double role in causing living cells to die, by triggering the pro-apoptotic routes, e.g., cysteine protease response to dehydration 21 - RD21, hydroxyacylglutathione hydrolase 2 (GLY2) as well as by inhibiting the pro-survival factors including SET protein (which acts as an inhibitor of p53 acetylation and blocks both p53-mediated cell cycle arrest and apoptosis after stress) or luminal binding protein 1 and 2 (BiP1 and BiP2) whose overexpression increased cell tolerance to endoplasmic reticulum stress as shown in tobacco protoplast (Leborgne-Castel et al., 1999; Martínez-Fábregas et al., 2014).

To reduce the negative impact of various stresses, including Pb pollution, the best solution may be biostimulators, which improve plant tolerance and protect them against harmful factors. Among many different protective substances naturally occurring in plants, melatonin (*N*-acetyl-5-methoxytryptamine) seems to have great biostimulatory potential (Janas and Posmyk, 2013). Melatonin has been detected in numerous plant species (Reiter et al., 2015). This indoleamine is a broad-spectral antioxidant. It stimulates antioxidant enzymes and synthesis of glutathione, and activates other antioxidants (Bałabusta et al., 2016). It also increases the efficiency of mitochondrial electron transport chain thereby decreasing electron leakage thus limiting free radical generation (Kładna et al., 2003; Rodriguez et al., 2004; Leon et al., 2005; Tan et al., 2007; Reiter et al., 2015; Bałabusta et al., 2016). Moreover, the work of Galano et al. (2013), Tan et al. (2014) and Kołodziejczyk et al. (2015) indicated that the melatonin metabolites, e.g., cyclic-3-hydroxymelatonin, 2-hydroxylmelatonin and especially *N1*-acetyl-*N2*-formyl-5-methoxykynu-ramine (AFMK) also possessed antioxidant activity. These facts, together with melatonin small size makes it particularly capable of translocating easily between cell compartments and of protecting cell structures against excessive ROS.

Melatonin is also useful to protect plants against heavy metal-induced stresses (Tan et al., 2007). Presowing melatonin treated seeds eliminated the toxic effects of copper ions in *Brassica oleracea* rubrum during germination (Posmyk et al., 2008) and zinc sulfate in *Hordeum vulgare* L. roots (Arnao and Hernández-Ruiz, 2009). Relatively little is known about the specific mechanisms of melatonin action at the subcellular level in plants. Lei et al. (2004) showed that pretreatment with melatonin of carrot suspension cells attenuated cell damage caused by cold exposure.

Studying the molecular pathways of PCD in whole plants introduces many difficulties, because it often occurs in a small number of directly stress-affected cells (McCabe and Leaver, 2000). Thus, for analysis of cytotoxic effects, cell lines are of particular suitable. *Nicotiana tabacum* L. cv Bright Yellow 2 (BY-2) suspension cells are fast growing higher plant cells, which provide an excellent model for examining plant physiology, biochemistry and molecular biology (Nagata et al., 1992). They allow research both at the level of a single cell and in its compartments. The objective of the present study was to determine if pretreatment of a suspension *Nicotiana tabacum* BY-2 cells with melatonin inhibits Pb-induced PCD. The findings show that melatonin significantly limited the negative effects of this heavy metal and acted as a biostimulating, pro-survival factor.

## Materials and Methods

### Plant Material

Sterile suspensions of *in vitro* cell cultures of *Nicotiana tabacum*, L. cv Bright Yellow 2 (BY 2) were used. The cells were cultivated in Linsmaier and Skoog (1965) basal medium (LS) supplemented with 30 g l^-1^ sucrose, 0.2 mg l^-1^ 2,4-dichlorophenoxyacetic acid (2,4-D; synthetic auxin), 1 mg l^-1^ thiamine, 0.1 g l^-1^ myo-inositol and 10^-2^ M KH_2_PO_4_. The initial pH of the medium was established as 5.3.

### Cell Culture and Growth Conditions

BY-2 suspended cells were routinely propagated and cultured at 25°C. From the stationary growth phase (day 7th) of the base culture, 2 ml of cell suspension were passaged into the fresh LS medium as a control (C) and LS with 200 nM melatonin (MEL). The optimal dose of melatonin was chosen experimentally. In the middle of the logarithmic phase of growth (day 4th) Pb(NO_3_)_2_ was added to LS (Pb) and LS with melatonin (MEL + Pb) media to the final Pb^2+^concentration 15 μM. Thus, the experiments were performed in four variants: (i) C: BY-2 cells cultured under optimal conditions on LS medium, (ii) MEL: BY-2 cells cultured on LS medium supplemented with melatonin from the start of new culture; (iii) Pb: BY-2 cells cultured on LS medium with Pb^2+^ added on the 4th day of culture and (iv) MEL + Pb: BY-2 cells cultured on LS medium with melatonin added from the start of culture and stressed with Pb^2+^ added on the 4th day of culture. The cultures were maintained to the 7th day (stationary phase of the control cell growth). The applied concentration of lead was chosen after measurement of LC_50_ on the 7th day.

### Determination of Cell Growth and Viability

The cell number was determined with the use of a Fuchs-Rosenthal haemocytometer under a light microscope Olimpus CX-31 equipped with MicroScan v.15. digital system of image analysis; additionally the number of dead cells was assessed after selective staining with methylene blue. Living cells do not take up the stain and retain their natural color whereas damaged cells are stained blue as they are unable to keep the methylene blue from penetrating their membranes. The number of cells and their viability were analyzed every experimental day.

### Melatonin Determination

Melatonin was extracted according to the modified methods of Guerrero et al. (2001) and Hernandez-Ruiz et al. (2004). Its concentration was measured during *lag, log* and the stationary phases of growth. After filtration and separation of the cells from the medium concentrations of melatonin in the extracts were determined using high-performance liquid chromatography (HPLC-MS/MS). For extraction, 5 g of fresh weight of the cells was homogenized with 5 mL of 50 mM sodium phosphate buffer (pH 8.0) containing 1 mM EDTA and 5 μM butylated hydroxytoluene (BHT) as an antioxidant. The homogenate was maintained for 15 h at room temperature in darkness with minimal shaking, in order to ensure complete extraction of melatonin.

The homogenate was centrifuged at 15000 *g* for 10 min at 5°C. Initial purification consisted in two steps by solvent-partitioning using ethyl acetate and 50 mM sodium phosphate buffer (first at pH 8.0 and second at pH 3.0). The two organic phases were evaporated together under vacuum. Dry residue was re-dissolved in 1 mL of mobile phase, filtered through Supelco ISO-Disc filters (PTEF-4 – 2.4 mm × 0.2 m; Supelko, Bellefonte, PA, United States), and frozen at -70°C until HPLC-MS analysis. The purified extract was subjected to HPLC-MS/MS analysis using an Agilent 1200 LC System coupled with AB Sciex 3200 QTRAP mass detector equipped with TurboSpray Ion Source (ESI). Each sample was injected onto Agilent SB-C18 column.

### Assay of Cell Death by Fluorescent Microscopy

Detection and verification of cell death in the suspension of cells were carried out according to Byczkowska et al. (2013) procedure: (1) 0.5 mL of the culture medium with 0.5 mL of the appropriate cell suspension was supplemented with 0.5 mL of 0.02 M phosphate buffer pH 7.4 (PHB). (2) The cells were stained with the AO/EB mixture containing 50 μg cm^-3^of acridine orange and 50 μg cm^-3^ of ethidium bromide in PHB. (3) Drops of cell suspension were immediately put on glass slides and analyzed for 5 min using fluorescent microscopy with a blue light excitation filter of the Optiphot-2 epi-fluorescence microscope (Nikon) equipped with a camera and Act-1 software (Precoptic, Poland) for fluorescent microscopy and preparation of microphotographs according to Byczkowska et al. (2013). AO/EB staining included the use of acridine orange which penetrates whole cells and stains the nuclei green and with ethidium bromide which dyes nuclei red and it is only absorbed by damaged cells with impaired cellular and nuclear membrane integrity. From the above data, a curve of the fluorescence intensity of nuclear chromatin after AO/EB staining was prepared, as described by Byczkowska et al. (2013). This scale allows the recognition of living, dying and death cells. Living cells have intact nuclei stained green, while dying cells have green–yellow, yellow, yellow–orange, or bright orange nuclei with slightly condensed or fragmented chromatin at the early stage of death whereas with condensed and fragmented chromatin at the late stage. Necrotic cells have structurally normal orange nuclei.

When the color is changed from green to red, values of fluorescence intensity of acridine orange and ethidium bromide increase (Byczkowska et al., 2013).

### Cell Fractionation

Fractionation of cells was performed using the digitonin method according to Ganju and Eastman (2003) with modification of Kobylińska et al. (2006). In all experimental variants the cells were washed twice with PBS and next permeabilized for 30 min in a buffer containing: 1 mM NaH_2_PO_4_, 8 mM Na_2_HPO_4_, 75 mM NaCl, 250 mM sucrose, digitonin (0.05% of cells weight), 20 μl/g cells 1 mM phenylmethylsulfonyl fluoride (proteases inhibitor), and cocktail of enzymes for cell wall lysis (CellLytic Sigma). Cell homogenate was obtained by centrifugation at 3000 *g* for 1 min. at 4°C to remove cell debris. The cleaned homogenate after centrifugation at 12000 ×*g* was divided into two fractions: the supernatant was removed as the cytosolic fraction and the pellet (mitochondrial fraction) was resuspended in the above buffer (without digitonin). To both fractions sufficient volumes of Laemmli sample buffer supplemented with 10% β-mercaptoethanol were added (Laemmli, 1970) and the mixtures were boiled for 5 min.

### Western Blot Analysis

Fractionated BY-2 cell lysates (50 μg of proteins) were electrophoretically separated by sodium dodecyl sulfate polyacrylamide gel electrophoresis (SDS-PAGE) on 15% gel (Laemmli, 1970) and transferred to Immobilon P^SQ^ at the voltage of 20 V overnight, at 4°C according to Towbin et al. (1979). After blocking in 3% non-fat dry milk in TBST (10 mM Tris-HCl, pH 7.5, 150 mM NaCl, 0.05% Tween-20) for 60 min, the membranes were incubated with primary antibodies specific to Cyt c in TBST in a cold room overnight. Subsequently, the membranes were washed several times in TBST and incubated with appropriate secondary antibodies conjugated with alkaline phosphatase (Sigma Chemical Co.) in TBS for 2 h at room temperature. Next the membranes were washed several times with TBST, and the proteins were visualized by incubation with the substrate solution (0.33 mg/ml of nitro blue tetrazolium, 0.17 mg/ml of 5-bromo-4-chloro-3-indolyl phosphate in 100 mM Tris-HCl, pH 9.5, 100 mM NaCl and 5 mM MgCl_2_), prepared according to Leary et al. (1983).

### DNA Isolation

DNA digestion was performed using the cetyl-trimethyl-amonnium bromide (CTAB) method previously described by Murray and Thompson (1980). BY-2 cells from *log* phase of growth (4th day, 4 h after Pb^2+^ addition) and from the beginning of the stationary phase (6th day, 48 h after Pb^2+^ addition) were frozen in liquid nitrogen and ground in a mortar to a fine powder. Then, CTAB buffer (100 mM Tris-HCl pH 8.0, 1,4 M NaCl, 20 mM EDTA and 2% CTAB) was added and extraction was performed for 30 min at 65°C. After immediate cooling on ice, DNA preparation was continued in the extraction mixture of chloroform/isoamyl alcohol (24:1) until a fine emulsion was created. The organic phase was separated from the aqueous phase by centrifugation at 12000 ×*g* for 15 min at 4°C. DNA was precipitated with isopropanol at -20°C, 20 min. The DNA precipitates were spun at 12000 ×*g* for 10 min at 4°C, washed two times in 70% ethanol and air dried. DNA pellets were dissolved in 100 μl TE buffer (10 mM Tris-HCl pH 7.5, and 1 mM EDTA) containing 10 μl 1% RNase A. RNA digestion was conducted 2 h at 37°C. Purity of the obtained DNA preparations was determined spectrophotometrically by analysis of the absorbance spectra in the range of 230–320 nm. The value of A_260/280_ within the limits of 1.8 – 2.0 was the criterion of DNA purity. Then, 5 μl of a loading buffer was added to each tube, and the DNA preparations were electrophoresed in 2% agarose gels and run at 5 V/cm. The gels were stained with ethidium bromide and visualized under ultraviolet (UV) light.

### Statistical Analysis

The data represent the means ± standard deviation (±SD). Each variant of culture was replicated three times and at least three independent samples were used for measurement. The data were analyzed using STATISTICA v.10.0_MR1_PL [StatSoft] software. One-way or two-way analysis of variance (ANOVA) and then the *post hoc* Duncan multiple range test was carried out to find the significant differences at *p* < 0.001 in each experiment.

## Results

### Cell Growth and Viability

**FIGURE 1:**
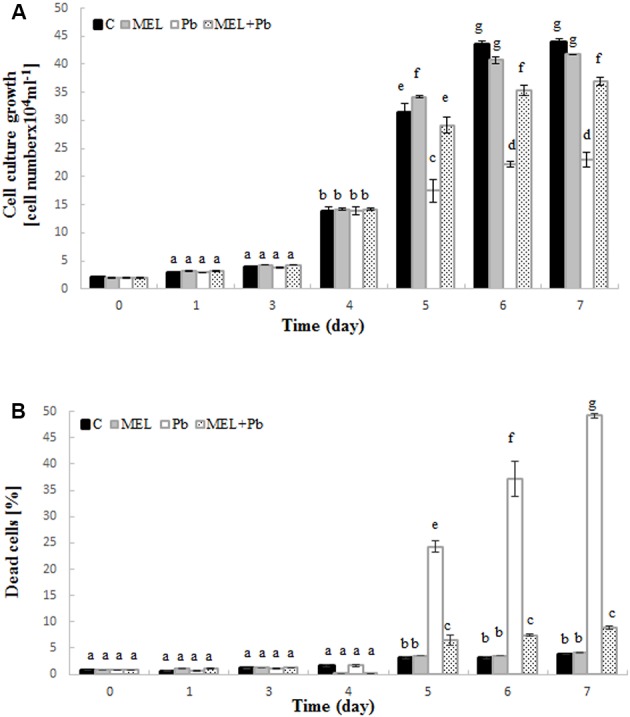
Level of cell growth **(A)** and mortality **(B)** of BY-2 tobacco cells in conducted experiments. C, BY-2 cells cultured on LS medium – the control variant; MEL, BY-2 cells cultured on LS medium with 200 nM melatonin added from the beginning of the culture; Pb, BY-2 cells cultured on LS medium with 15 μM Pb^2+^ added on the 4th day of the culture and MEL + Pb, BY-2 cells cultured on LS medium with melatonin added from the start of the culture and with Pb^2+^ added on the 4th day of culture. The results are expressed as mean values of 3 independent experiments ± SD. Two-way ANOVA and Duncan’s *post hoc* test were performed. The small letters next to the values show statistical significance *p* < 0.001. **(A)** Viability ANOVA results: Variant (C, MEL, Pb, MEL + Pb) *F*_(3;56)_ = 1189, *p* < 0.0001; Time (0, 1, 3, 4, 5, 6, 7) *F*_(6;56)_ = 11505, *p* < 0.0001; and interaction Variant × Time *F*_(18;56)_ = 270, *p* < 0.0001. **(B)** Mortality ANOVA results: Variant (C, MEL, Pb, MEL + Pb) *F*_(3;56)_ = 3207, *p* < 0.0001; Time (0, 1, 3, 4, 5, 6, 7) *F*_(6;56)_ = 1637, *p* < 0.0001; and interaction Variant × Time *F*_(18;56)_ = 756, *p* < 0.0001.

Preincubation with melatonin prior to Pb treatment protected tobacco suspension cells from death and improved cell proliferation. Cell growth intensity in C and MEL variants was similar during culture time. After Pb addition on the 4th day, a significant inhibition of tobacco cell proliferation was observed (**Figure 1A** – see the variants MEL + Pb and especially Pb). From the first day after heavy metal stress induction, proliferation of the MEL + Pb cells was about 40% higher in comparison to those treated with Pb but not primed with melatonin (Pb) (**Figure 1A**) this tendency was maintained throughout the duration of the Pb-stress.

The effects of melatonin pretreatment on viability of Pb-stressed tobacco suspension cells were verified in all experimental samples. Methylene blue staining documented the protective effect of melatonin against cell death induced by Pb. The mortality of cells exposed to Pb but preincubated with melatonin (MEL + Pb) was slightly higher than in C and MEL variants. Culture medium supplementation with melatonin did not result in cell death acceleration. The number of dead cells in the Pb exposed cells increased significantly and it was 24.2, 37.3, and 49.2% for the 1st, 2nd, and 3rd day after Pb application, respectively (**Figure 1B**). In contrast, mortality of MEL + Pb cells was about 80% lower than in the Pb cells (**Figure 1B**).

### Detection of Cell Death

**FIGURE 2:**
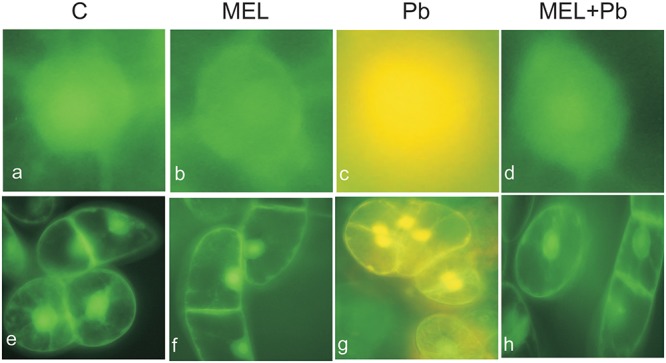
Micrographs of living and dying BY-2 nuclei **(a–d)** and whole cells **(e–h)** detected by AO/EB staining. *Green nuclei* of living cells **(a,b,d,e,f,h)**, *yellow nuclei* **(c,g)** of PCD-dying cells. C, BY-2 cells cultured on LS medium – the control variant; MEL, BY-2 cells cultured on LS medium with 200 nM melatonin added from the beginning of culture; Pb, BY-2 cells cultured on LS medium with 15 μM Pb^2+^ added on the 4th day of culture and MEL + Pb, BY-2 cells cultured on LS medium with melatonin added from the start of the culture and with Pb^2+^ added on the 4th day of culture. Micrographs were done 4 h after lead administration.

Fluorescence analyses after successive addition of AO/EB fluorochromes showed that after Pb treatment BY-2 cells died *via* PCD. Yellow and green-yellow nuclei with slightly condensed chromatin dominated among dying cells (**Figure 2**), but some yellow-stained nuclei with condensed chromatin were also observed. This observation indicates that already 4 h after Pb treatment, BY-2 cells underwent the initial stages of cell death. At the end of cell culture (the 7th day; the 3rd day after Pb stress) nuclei with dark orange chromatin were not found, indicating that necrotic type of cell death after Pb exposure in BY-2 tobacco cells was not detected.

**FIGURE 3:**
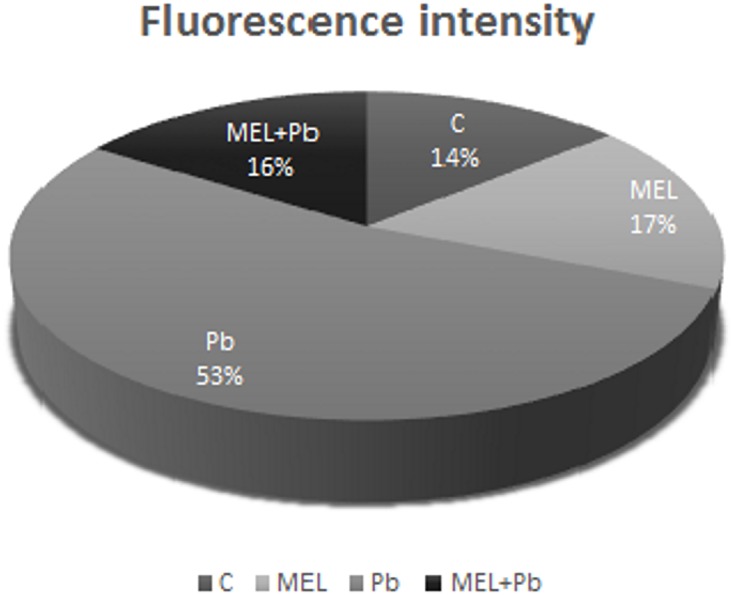
The fluorescence intensity of nuclear chromatin stained with AO/EB 4 h after lead administration. C, BY-2 cells cultured on LS medium – the control variant; MEL, BY-2 cells cultured on LS medium with 200 nM melatonin added from the beginning of culture; Pb, BY-2 cells cultured on LS medium with 15 μM Pb^2+^ added on the 4th day of culture and MEL + Pb, BY-2 cells cultured on LS medium with melatonin added from the start of the culture and with Pb^2+^ added on the 4th day of culture.

Unexpected effects were obtained for cells exposed to Pb but pre-incubated with melatonin (MEL + Pb). The fluorescence intensity of randomly selected nuclei for this treatment was estimated at 16% and it was similar to the Pb-untreated samples: 14 and 17% for control and melatonin treated cells respectively. In contrast fluorescence intensity in Pb variant increased to 53%, and was expressed as yellow/yellow–orange nuclei color (**Figure 3**).

### Profile of DNA Fragmentation

**FIGURE 4:**
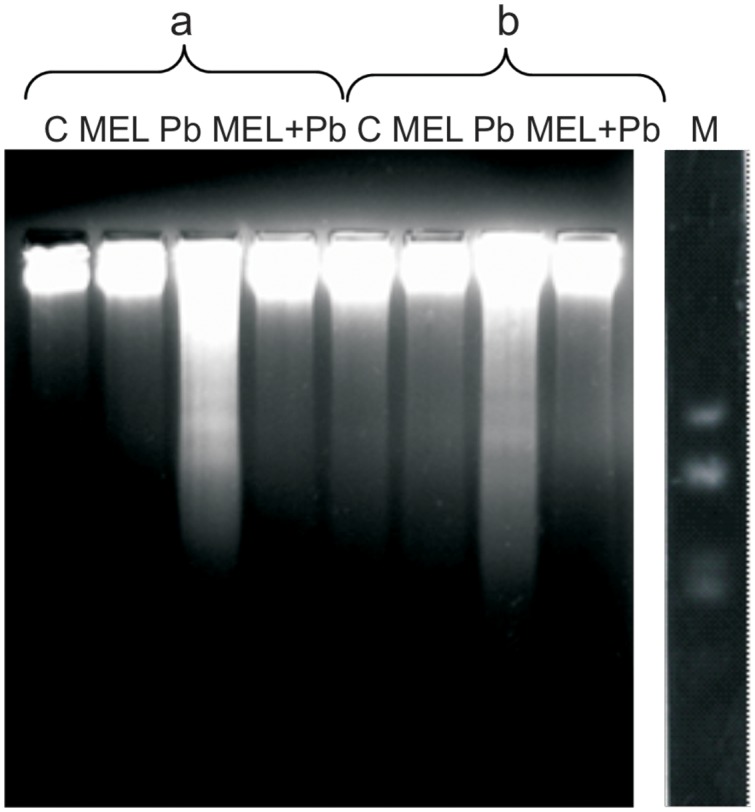
Induction of DNA fragmentation in BY-2 cells exposed to lead. C, BY-2 cells cultured on LS medium – the control variant; MEL, BY-2 cells cultured on LS medium with 200 nM melatonin added from the beginning of the culture; Pb, BY-2 cells cultured on LS medium with 15 μM Pb^2+^ added on the 4th day of the culture and MEL + Pb, BY-2 cells cultured on LS medium with melatonin added from the start of the culture and with Pb^2+^ added on the 4th day of culture. DNA was obtained from the cells: 4, on the 4th day – 4 h after lead administration and 6, on the 6th day – 2 days after lead administration. DNA was analyzed by 2% agarose gel electrophoresis; M, molecular weight marker (pUC 18DNA Hae III digest).

To gain insight into the mechanism of plant PCD induced by Pb, DNA fragmentation and release of Cyt c from mitochondria into cytosol was checked. We found an inhibitory effect of melatonin on DNA laddering, one of the hallmarks of PCD. **Figure 4** shows that DNA isolated from control and melatonin treated cells remained intact, whereas DNA from Pb samples exhibits significant fragmentation; this was more intensive 4 h after Pb stress then 2 days later (the 6th culture day, the 2nd day after lead treatment). The analyses showed that melatonin completely blocked/reversed the cytotoxic Pb influence and protected tobacco cells against DNA damage caused by the heavy metal.

### Cytochrome c Translocation

**FIGURE 5:**
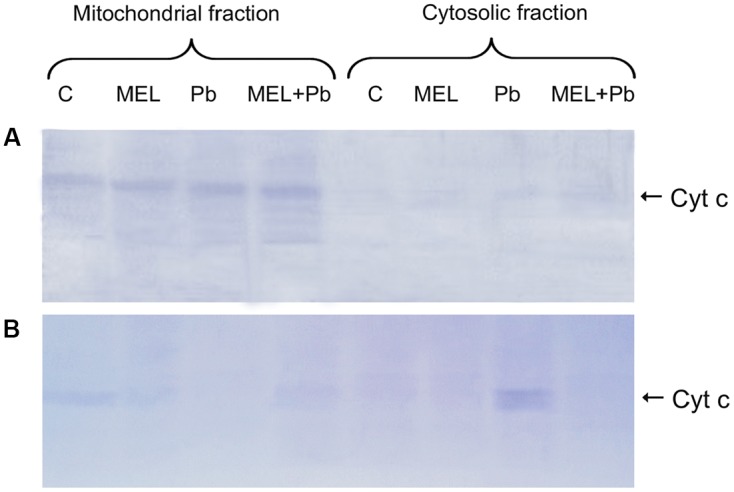
Expression of the cytochrome c protein. **(A)** the 4th day – 4 h after lead administration and **(B)** the 6th day – 2 days after lead administration. Lysates from untreated BY-2 cells (C), cells grown on the medium supplemented with melatonin (MEL) and cells exposed to lead without and with melatonin treatment (Pb and MEL + Pb, respectively).

To further confirm the protective action of melatonin, immunodetection of Cyt c in mitochondrial and cytosolic fractions was examined. There is growing evidence that in plants, as in mammals, translocation of Cyt c from mitochondria to cytosol plays an important role in PCD mediated events. Detection of this protein with an antibody recognizing whole Cyt c molecule was performed in mitochondrial pellet and cytosolic fractions of BY-2 cells in all experimental groups. Unexpected effects were obtained in the 4th hour after Pb treatment, despite DNA fragmentation at that time, Cyt c was detected only in the mitochondrial pellet, suggesting that Cyt c release from mitochondria to cytosol is a later stage of PCD and takes place independent on DNA damage (**Figure 5A**). Relatively low, but different levels of Cyt c accumulated in mitochondrial pellet of BY-2 cells in control (C) and melatonin-treated samples (MEL and MEL + Pb) on the 2nd day after Pb treatment (**Figure 5B**). Release of Cyt c from mitochondria into cytosol was observed after Pb exposure where translocation of Cyt c was accompanied by almost complete disappearance of this protein from the mitochondria and its accumulation in the cytosolic fraction. MEL + Pb samples were deficient of Cyt c in cytosol, similar to Pb-untreated cells. This cytological and molecular evidence demonstrates that melatonin preincubation protects tobacco suspension cells from Pb-induced PCD.

### Content and Cellular Localization of $O_{2}^{\cdot -}$

**FIGURE 6:**
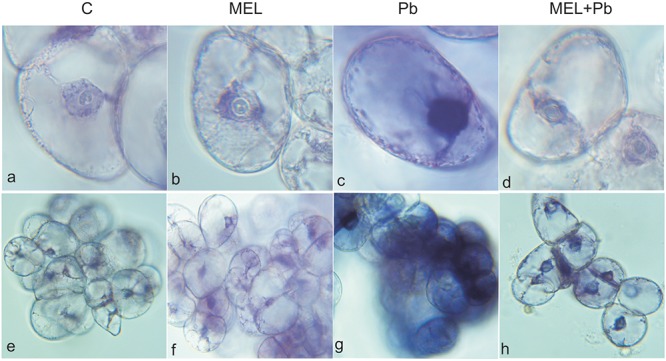
Superoxide anions detected by NBT staining in tobacco BY-2 cells. The cells were stained 4 h after lead administration. C, BY-2 cells cultured on LS medium – the control variant; MEL, BY-2 cells cultured on LS medium with 200 nM melatonin added from the beginning of the culture; Pb, BY-2 cells cultured on LS medium with 15 μM Pb^2+^ added on the 4th day of culture and MEL + Pb, BY-2 cells cultured on LS medium with melatonin added from the start of the culture and with Pb^2+^ added on the 4th day of culture. BY-2 cells zoomed × 1000 **(a–d)** and × 100 **(e–h)**.

To investigate whether the observed a Cyt c release is related to Pb-induced ROS production, we measured *in situ* accumulation of $O_{2}^{\cdot -}$ *via* the nitro blue tetrazolium reducing (NBT) test. In the non-stressed cells (C, MEL), few formazan precipitants were apparent indicating the physiological origin of ROS. Cytological analyses of $O_{2}^{\cdot -}$ production in tobacco suspension cells demonstrated, abundant formazan deposits after Pb exposure. They were especially visible in the boundary cytoplasm and in nuclei which appeared almost black (**Figure 6**). In contrast, in the MEL + Pb samples the amounts of formazan precipitants was similar to that in the control, confirming a reduction in $O_{2}^{\cdot -}$ by melatonin.

### Melatonin Content in BY-2 Cells

To test if the protective function of melatonin treatment is related to its uptake from the environment and accumulation in cells, the content of this indoleamine in cell lysates was determined at the main experimental points, i.e., in the *lag, log* and stationary phases of growth. Generally, BY-2 tobacco cells have low melatonin levels (**Table 1**). In the non-melatonin-primed cells (C and Pb), melatonin increased from zero (the 1st day of culture) to ∼1 ng/g_FW_ (on the last day). Tobacco suspension cells synthesize endogenous melatonin, but at an extremely low level in comparison with the melatonin-treated cells (MEL and MEL + Pb). In addition, in cells under Pb stress the endogenous level of this indoleamine in comparison to the control (C) cells was about 30% lower at the end of the culture period (7th day). Concentrations of melatonin significantly increased in the cells during the period of melatonin treatment (**Table 1**). The data indicate that BY-2 cells absorbed melatonin from the medium. Interestingly, MEL + Pb cells absorbed 20% more than MEL-treated cells alone.

**Table 1:** Melatonin concentration (ngMEL/gFW) in homogenates of BY-2 cells in crucial points of conducted experiments.

Day of culture → Variant	1	4 ^1)^	7
C	0.00 ± 0.00	0.72 *a* ± 0.01	0.94 *a* ± 0.05^∗^
MEL	6.70 *b* ± 0.24	15.18 *c* ± 2.10	34.92 *d* ± 4.00
Pb	–	–	0.65 *a* ± 0.24^∗∗^
MEL + Pb	–	–	41.31 *e* ± 3.28

*HPLC-MS measurements was performed: 1st day – during lag phase; 4th day after passaging – during log phase, ^1)^this day was also chosen as the start of Pb-stress; and 7th day – during stationary phase of growth (variants C and MEL) and when Pb-stress symptoms should be detectible (variants Pb and MEL + Pb). Experimental BY-2 cell were cultured according to following variants: control (C) – cells cultured on full LS medium, (MEL) – cell cultured on melatonin-supplemented medium, (Pb) – cells cultured with addition of lead alone and with pretreatment with melatonin (MEL + Pb). The results are expressed as mean values of 3–4 measurements ± SD. Two-way ANOVA and Duncan’s post hoc test were performed. The small letters next to the values show statistical significance p < 0.0001. Melatonin ANOVA results: Variant (C, MEL, Pb, MEL + Pb) F_(3;24)_ = 440, p < 0.0001; Time (1, 4, 7) F_(2;24)_ = 317, p < 0.0001; and interaction Variant × Time F_(6;24)_ = 100, p < 0.0001. In a case of 7^th^ day C and Pb variant additionally Student’s t-test were performed and the statistically significant difference between this two values was determined at p < 0.05 (marked by stars).*

## Discussion

Melatonin was discovered in the plant kingdom in Dubbels et al. (1995), Hattori et al. (1995) and significant progress has been made in defining its multiple roles in plants. Many researchers underline the fact that among its various roles, its antioxidant effectiveness and free radical scavenging ability, that protect plants against oxidative stress and alleviate or counteract cell damage, are crucial to plant physiology as in animals (Reiter et al., 1997; Tian et al., 2001). Melatonin is widely present in many higher plants (Arnao and Hernandez-Ruiz, 2015; Reiter et al., 2015). Elevated levels of melatonin protect plants against water and soil pollutants by acting as a direct free radical scavenger (Tan et al., 2007; Manchester et al., 2015) and/or as an indirect antioxidant stimulating antioxidant enzymes (Rodriguez et al., 2004; Bałabusta et al., 2016). Melatonin metabolites also posses antioxidant properties and they act in synergy with other antioxidants, such as ascorbic acid, glutathione, etc. (Gitto et al., 2001; Arnao and Hernández-Ruiz, 2009; Kołodziejczyk et al., 2015). Melatonin protects plant tissues and organs, particularly reproductive tissues, fruit and germ tissues of the seeds, from secondary oxidative stress caused by unfavorable environmental conditions, such as drought, salinity, cold, heat, ultraviolet light and ozone (Van Tassel et al., 2001; Dawood and El-Awadi, 2015; Reiter et al., 2015). The data indicate that exogenously applied melatonin also acts as plant biostimulator especially under suboptimal environmental conditions (Posmyk et al., 2008, 2009; Janas and Posmyk, 2013; Kołodziejczyk et al., 2016).

There is growing evidence that in plants melatonin action is associated with its antioxidant properties. Moreover melatonin as the antiapoptotic factor is well documented in various animal cells, but not in plants (Reiter et al., 1997, 2015). Thus, the aim of our investigation was to check melatonin influence on heavy metal-induced cell death in tobacco suspension cultures. There is rather little information on the mechanisms by which melatonin prevents plant cells from dying after Pb exposure.

Presented studies were preceded by analyses of influence the different melatonin concentrations as well as Pb, on BY-2 suspension cells (data not shown). The range of tested melatonin concentrations was 100–1000 nM, whereas Pb 0.5–50 μM. The concentration of Pb applied in presented experiments was chosen on the basis of measurement of LC50 on the 7th day of BY-2 cell cultivation. Our preliminary studies revealed that exogenous melatonin has ambiguous effects on BY-2 cells: it is an effective biostimulator when applied in concentration below 300 nM, but in excessive doses (above 300 nM) it significantly decreased both BY-2 cell proliferation and viability. This agrees with the previous publications of our team, where we also observed different effects of melatonin treatments dependent on the dosage used (Posmyk et al., 2008, 2009; Janas and Posmyk, 2013). This indicates, that melatonin, despite its potentially positive properties (e.g., antioxidative), can not be considered as being always protective since high concentration may have harmful side effects. The choice of dosage is crucial for the positive effects to be realized; in BY-2 cells melatonin was protective in the range 100–300 nM.

Our preliminary experiments led us to determine the optimal melatonin dose as 200 nM that stimulated proliferation of BY-2 cells and nearly completely reversed effects of Pb-stress. Thus, initially cell proliferation and viability during *lag, log* and stationary phases of growth both under optimal (C and MEL) as well as under heavy metal stress condition (Pb and MEL + Pb) were determined. The positive effects of preincubation of BY-2 cells with melatonin were visible during and after Pb-stress which suggests that melatonin at a concentration 200 nM fortifies cells against potentially stress conditions even before they appear. Proliferation of cells pretreated with melatonin but exposed to Pb (MEL + Pb) were only slightly worse than of the unstressed cells (C and MEL) whereas in the Pb-stressed cells the level of proliferation was more than 50% lower in comparison to the control. The protective role of melatonin against cell death was clearly visible in the cell mortality analyses during Pb-stress. The number of dead cells in the Pb-treated cells increased drastically while unexpectedly in the MEL + Pb variant BY-2 cell viability was about 80% higher than in the Pb samples (**Figure 1B**). The melatonin ability to prevent cell death was confirmed by the studies of fluorescence intensity of nuclear chromatin stained with AO/EB. In our experiments morphological changes in the nuclei of the Pb exposed cells, i.e., chromatin condensation, green–yellow and yellow color of nuclei, was shown (**Figures 2**, **3**).

Plant cells that undergo PCD exhibit many of the same morphological characteristics as cells undergoing PCD in mammals and *Caenorhabditis elegans* including intensified formation of vesicles, cytoplasmic condensation, nuclear condensation, DNA fragmentation and chromatin condensation leading to DNA laddering as well as translocation of Cyt c (Balk et al., 2003; Lord and Gunawardena, 2012). Our results showed that in BY-2 cells DNA fragmentation appeared already at 4 h after Pb application and it persisted until the 2nd day after Pb application. In electrophoregrams of DNA samples isolated from the Pb-exposed cells extensive oligonucleosomal fragmentation caused by this heavy metal was observed, which was not noted in untreated cells (C and MEL). In the MEL + Pb treated cells melatonin completely blocked DNA laddering. Our results are in line with the studies of Lei et al. (2004) who showed that melatonin increased tolerance to cold in carrot suspension cells and protected their DNA against damage. Similar beneficial effects caused by exogenous melatonin were observed by Posmyk et al. (2008) in red cabbage seedlings subjected to copper stress and in cucumber seedlings subjected to chilling stress (Posmyk et al., 2009).

The data presented suggest that pre-incubation of BY-2 cells with melatonin limits the toxicity of Pb and protects cells against death. We used the plant model system to pinpoint the PCD phase when melatonin may act. It has been reported that release of Cyt c from mitochondrial intermembrane space to cytosol is a conserved pathway of PCD and it has been noted in many systems (Martínez-Fábregas et al., 2013). In plants, this issue has been poorly investigated and the mechanisms of Cyt c release, its role in determining cell death are still controversial. Vacca et al. (2006) reported that heat shock triggered Cyt c translocation but Cyt c was degraded en route to cell death. Moreover, Cyt c release is linked to ROS burst and strictly depends on ROS production, and it identifies the early phase of cell death. However, our Western blot analyses did not reveal translocation of Cyt c from mitochondria to cytosol 4 h after the heavy metal stress rather this occured much later (the 6th day of culture, the 2nd day after lead administration). Bolduc and Brisson (2002) as well as Kawai-Yamada et al. (2004) reported that oxidative metabolism leading to generation of ROS was one of the earliest events in PCD induced by biotic or abiotic stress in tobacco plants. Thus, this suggests that high levels of ROS mediate the signaling cascade for defenceive gene induction, e.g., *hsp, lea*, *cor* (Vincour and Altman, 2005). In our studies, ROS production and DNA laddering were the early features of PCD and translocation of Cyt c was independent of them. We observed DNA fragmentation before Cyt c translocation, which seems to support the hypothesis of Collazo et al. (2006) that NO/ROS ratio may induce a set of defense responses including cleavage of an inhibitor of caspase-activated DNAse (ICAD) (Krishnamurthy et al., 2000; Collazo et al., 2006). Although the presence of caspases in plants is debated, cysteine protease activity has been reported to be induced in plant systems undergoing cell death (Lam and del Pozo, 2000; Bozhkov et al., 2004). These proteases might function in a plant proteolytic network leading to disconnection of ICAD from DNAse (CAD) and fragmentation of DNA. Moreover, it would confirm our observations that in MEL + Pb cells, despite Pb application, DNA was not cleaved since this indoleamine is a highly effective antioxidant and it blocked caspase-like signaling leading to activation of CAD. Furthermore, our results revealed accumulation of superoxide radical in Pb exposed cells at the 4th hour after Pb treatment with only a slight detection in the control (C) and melatonin treated cells (MEL, MEL + Pb) (**Figure 6**). Thus, our finding are in line with the data of Vacca et al. (2006) who showed that ROS scavenging inhibited Cyt c release. Jou et al. (2004) documented that in rat brain astrocytes melatonin inhibited opening of mitochondrial permeability transition pore (MPT) and blocked MPT-dependent Cyt c release.

To test whether tobacco cells synthesize endogenous melatonin, and/or are capable of active absorption exogenous melatonin from the environment, the contents of this indoleamine in cell lysates were determined at the crucial points of the experiments, i.e., *lag*, *log* and stationary phase of growth.

Our results indicated that tobacco BY-2 cells are able to synthesize small amounts of this indoleamine depending on the phase of growth (its endogenous level increased slightly during experiment) as well as to absorb it actively from the medium (**Table 1**). It is known that biosynthesis and metabolism of this indoleamine are affected and modified by environmental conditions (i.e., stresses), and melatonin levels change during plant ontogenesis (Okazaki and Ezura, 2009). Elevated melatonin synthesis is often combined with the plant defense strategy because generally it was noticed that various plant species rich in melatonin had greater capacity for stress tolerance (Park et al., 2013; Bajwa et al., 2014; Zhang et al., 2015).

Availability of exogenous melatonin allowed BY-2 cells to take up its large quantities throughout the culture period (**Table 1**). Similar results were observed by Kołodziejczyk et al. (2015) in the case of cucumber and corn seeds which were primed with exogenous melatonin – they absorbed quantities of this indoleamine proportional to its concentration applied during priming (Kołodziejczyk et al., 2015). Interestingly, under unfavorable conditions, Pb-stressed cells (MEL + Pb) absorbed melatonin near 20% more intensively in comparison to the unstressed melatonin treated cells (MEL). Although, it is probably not a natural defense strategy of *Nicotiana tabacum* cells since endogenous melatonin content did not measurably increase in BY-2 cells under Pb stress conditions (**Table 1**). These results suggest that BY-2 cells readily absorb and use the accessed exogenous melatonin to counteract stress-induced damage. Survival of plants in polluted environments largely depends on their ability to sequester and/or detoxify toxic substances such as Pb. Tan et al. (2007) also found that melatonin was effective in preventing the death of pea plants grown in soil contaminated with copper.

We have shown that although tobacco is not a plant rich in endogenous melatonin, it is able to use it from an exogenous source as a potential effective factor for improving its stress defenses. This fact once again confirms the practical use of melatonin as a plant biostimulator (Janas and Posmyk, 2013; Kołodziejczyk and Posmyk, 2016; Nawaz et al., 2016). Moreover, we have presented novel findings concerning a decrease of mitochondrial Cyt c release together with limited DNA degradation, which suggest that the protection mechanism of melatonin is not only *via* limitation of secondary oxidative stress, but also *via* counteraction against PCD. In conclusion, melatonin has multiple actions as a factor fortifying cells against potential, harmful conditions.

## Author Contributions

AK: work conception, all experiments concerning *Nicotiana tabacum* BY-2 suspension cells realization, data acquisition and analysis, drafting of the manuscript. RR: research consultation/discussion, manuscript revision: language and editorial corrections. MP: methodological consultant, statistical calculations, data analysis and interpretation, manuscript revision.

## Conflict of Interest Statement

The authors declare that the research was conducted in the absence of any commercial or financial relationships that could be construed as a potential conflict of interest.
